# Editorial: Neuroinflammation, neurodegeneration and metabolic disease: from molecular mechanisms to therapeutic innovation

**DOI:** 10.3389/fneur.2024.1478550

**Published:** 2024-08-28

**Authors:** Mohammed Al-Onaizi, Ayman ElAli, Fawaz Alzaid

**Affiliations:** ^1^Department of Anatomy, Faculty of Medicine, Kuwait University, Kuwait City, Kuwait; ^2^Dasman Diabetes Institute, Kuwait City, Kuwait; ^3^Neuroscience Axis, Research Center of CHU de Québec, Université Laval, Québec City, QC, Canada; ^4^Department of Psychiatry and Neuroscience, Faculty of Medicine, Université Laval, Québec City, QC, Canada; ^5^INSERM UMR-S1151, CNRS UMR-S8253, Université Paris Cité, Institut Necker Enfants Malades, Paris, France

**Keywords:** neuroinflammation, neurodegeneration, metabolic disease, microglia, parkinson's disease

The nervous system dynamically communicates and interacts with each tissue and organ system to regulate various critical physiological processes. The cellular components of these systems require a tightly functional and adaptable metabolism to meet their physiological needs. Inflammation, and more specifically neuroinflammation, is common in the development and progression of neurological and metabolic diseases.

**Figure 1:**
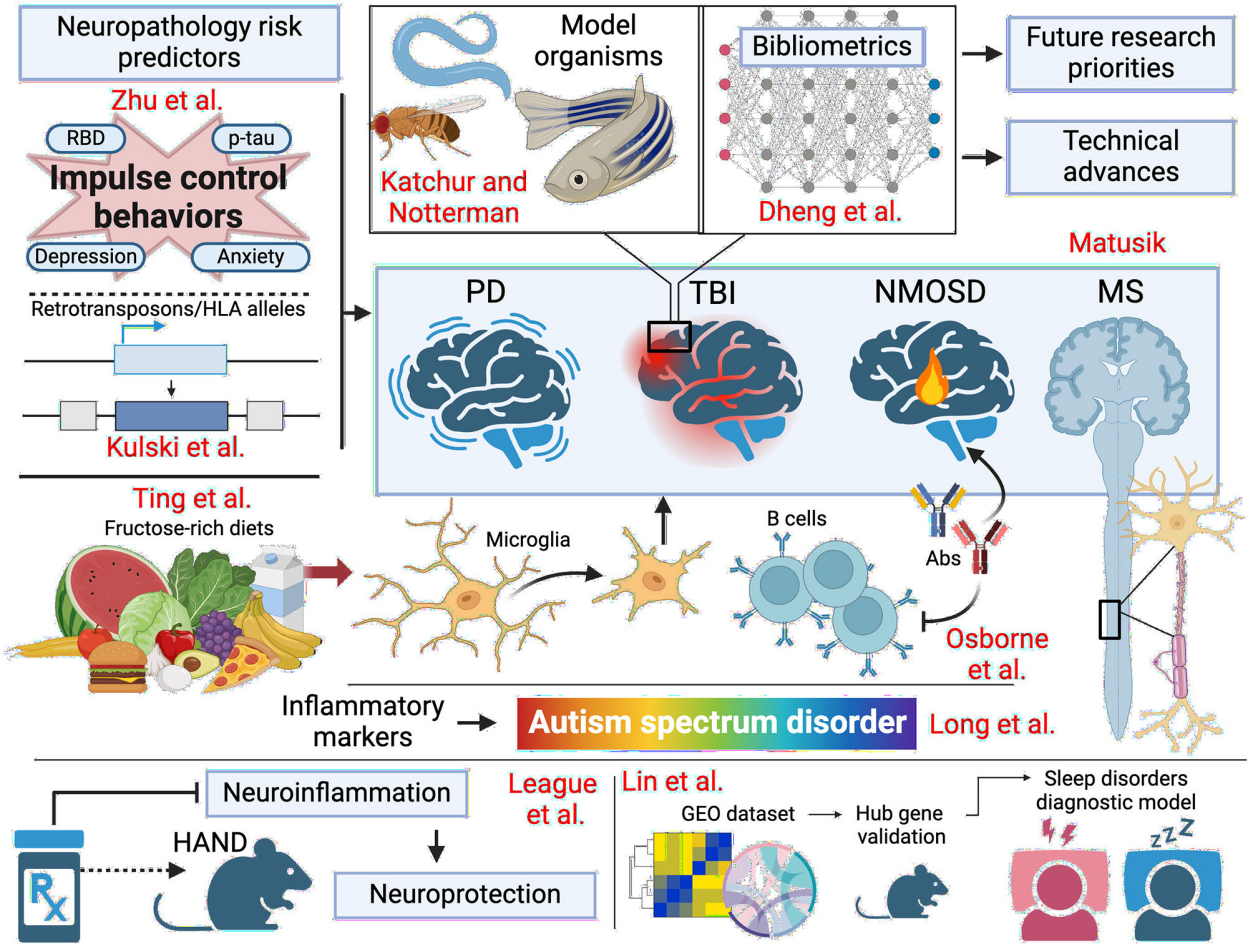
Schematic summary of the subjects covered by the articles contributing to this Frontiers in Neurology Research Topic “*Neuroinflammation, neurodegeneration and metabolic disease: from molecular mechanisms to therapeutic innovation*.” PD, Parkinson's disease; TBI, traumatic brain injury; NMOSD, neuromyelitis optica spectrum disorder; MS, multiple sclerosis; HAND, HIV-associated neurocognitive disorders; GEO, gene expression omnibus. Created with Biorender.com.

This Research Topic features recent studies that span the domains of neuroinflammation, neurodegeneration, and metabolic disease. We navigate through research at multiple levels, from longitudinal cohorts to clinical trials, bibliometric analyses, basic science, and reviews, to further our understanding of the intersection of neurological and metabolic diseases through the topic's focus areas. As we highlight novel genetic markers, predictive indicators, and the influence of diet on neuronal integrity, we have set a theme for current and future priority research areas in neurology. We aimed to highlight the breadth of manifestations of neuroinflammation in neurodegenerative and metabolic diseases. The summary schematic in Figure 1 illustrates the focus of the contributed articles.

Zhu et al. carried out a study based on data from the Parkinson's Progression Markers Initiative (PPMI) (1), to assess impulse control behavior (ICB) in early Parkinson's disease (PD). They reported that ICBs increase during the early stages of PD and that anxiety, rapid eye movement sleep behavior disorder, and p-tau levels in cerebrospinal fluid are predictors of the incident development of ICBs in early PD (Zhu et al.).

Further in the context of PD, another PPMI-based study by Kulski et al. investigated genetic and transcriptomic features in 1,521 individuals to assess the genotypes of eight classical HLA class I and II genes and the DRB3/4/5 haplotypes. They identified significant differences in HLA alleles and SVA (SINE-VNTR-Alu) retrotransposon insertions between PD patients and healthy controls, highlighting the influence of these genetic elements on PD progression and the immune response.

Moving to traumatic brain injury (TBI), Deng et al. conducted a bibliometric analysis of the role of stem cells in TBI recovery. Analyzing literature from the past 24 years, including 459 articles from 45 countries, the authors suggested that “immunomodulation” and “cellular therapy” are among the research hotspots in TBI, and that “exosomes,” “neuroinflammation,” and “microglia” are essential future research directions (Deng et al.).

Katchur and Notterman provided a topical review of non-mammalian models for studying the long-term effects of TBI and repetitive TBIs. These include sea lamprey, zebrafish and others, which are advantageous for their genetic tractability, reduced cost, and ethical considerations. These models are useful for mechanistic investigations of neurodegeneration associated with TBI, offering viable alternatives (Katchur and Notterman).

In autoimmune conditions, Osborne et al. carried out a retrospective study of patients with neuromyelitis optica spectrum disorder (NMOSD) who transitioned from rituximab to inebilizumab. Remarkably, 71.4% of patients experienced relapses during rituximab therapy, whereas no relapses were observed with inebilizumab for an average of 19.3 months. This suggests the effectiveness and safety of inebilizumab for NMOSD patients (Osborne et al.).

Matusik's work on multiple sclerosis (MS) assessed body mass index (BMI) and bioelectrical impedance analysis (BIA) in 176 MS patients. The BIA found a higher prevalence of overfatness compared to overweight by BMI, and BMI underestimated fat mass, especially in those with moderate disability. BIA correlated better with abdominal obesity and disability status. BIA has been shown to be superior for assessing nutritional status in MS patients (Matusik).

Moving further into the realm of immunity, Long et al. investigated circulating inflammatory factors in autism spectrum disorders (ASD). Two-sample bidirectional Mendelian randomization (MR) revealed that certain inflammatory factors (e.g., natural killer cell receptor 2B4) are positively associated with ASD, while others, like interleukin-7, are inversely associated. The authors suggested that inflammatory factors may indicate immunologic dysfunction in ASD.

In a mechanistic study of HIV-associated neurocognitive disorders (HAND), League et al. explored the potential of the monoacylglycerol lipase (MAGL) inhibitor MJN110 to mitigate Tat-induced neuroinflammation. In female Tat transgenic mice, MJN110 protected against neuroinflammatory hallmarks (e.g., dendritic injury) without altering behavior. The findings also show the neuroprotection by MJN110 is achieved without cannabimimetic behavioral effects (League et al.).

Next, Lin et al. investigated the mechanisms of sleep disorder (SD) by analyzing public datasets with advanced bioinformatics tools (e.g., LASSO, PPI networks). Differentially expressed genes were enriched in immune activity, stress response, and neural regulation, with elevated T cell levels found in SD patients. Hub genes were identified (e.g., IPO9, RAP2A), and then validated, and a diagnostic model using these genes demonstrated high accuracy.

Finally, and at the heart of this Research Topic, Ting provided a mini-review of a relevant area of nutrition and neurological health. Studies suggest that fructose may alter microglial function in the brain through metabolic reprogramming. This may influence activation and inflammation, increasing the risk of neurological dysfunction. Ting's review summarized the current findings and suggested directions for future research.

In conclusion, the collection of articles presented in this Research Topic not only underscores the multifaceted nature of neuroinflammation at the crossroads of neurological and metabolic diseases, but also provides insights into its understanding. From genetic landscapes to the metabolic triggers of neuroinflammatory responses, the insights offered represent a leap toward precision medicine. The therapeutic potential unlocked by the transition from rituximab to inebilizumab in treating NOMSD further exemplifies the advancements possible when innovative research converges with clinical application. By continuing along the path of converging biological domains and regrouping research at multiple levels, from clinical trials to bibliometrics, we hope to expand the range of promising areas for future research.
